# The Scent of Intimacy: Exploring the Associations Between Intimacy, Disgust, and Olfactory Ability

**DOI:** 10.1007/s10508-025-03145-y

**Published:** 2025-05-27

**Authors:** Ellen L. Murphy, Fiona E. Wylie, Mehmet K. Mahmut

**Affiliations:** Food, Flavour, and Fragrance Lab, School of Psychological Sciences, Macquarie University, Sydney, 2109 Australia

**Keywords:** Intimacy, Olfactory ability, Disgust sensitivity, Odor identification

## Abstract

Intimacy is a fundamental aspect of romantic relationships, yet its underlying sensory mechanisms remain understudied. Research has suggested that experiences of intimacy are related to both olfaction and disgust. For example, studies indicate greater olfactory ability is associated with fulfilling relationships, while heightened disgust sensitivity can hinder sexual arousal and interpersonal affiliation. However, no study has investigated intimacy, olfaction, and disgust together. To investigate this, 74 participants aged 17–56 years (*M* = 22.6, *SD* = 7.1), completed self-report surveys assessing relationship intimacy and sensitivity to pathogen, moral, and sexual disgust. Participants also completed a short form of the *Sniffin’ Sticks* identification test. Results revealed emotional and intellectual intimacy shared a significant weak positive association with olfactory ability, and no significant relationship with any form of disgust. Physical intimacy had a significant weak positive relationship with pathogen and sexual disgust, but not olfactory ability or moral disgust. Social intimacy had a significant positive moderate relationship with only pathogen disgust. Findings highlight the multifactorial nature of relationships and the importance of exploring different factors that may relate to intimacy.

## Introduction

Attaining an intimate, secure relationship with a romantic partner is regarded as a universal human need and crucial milestone (Erikson, 1959; Lafontaine et al., 2017). However, an increasing portion of the population report feeling lonely and disconnected, particularly in the aftermath of the COVID-19 pandemic (Patulny & Bower, 2022). Despite findings that both body odor and disgust sensitivity relate to intimacy (Granqvist et al., 2019; Zsok et al., 2017), no study has yet combined these factors. Understanding the relationship between these factors may assist in our broader understanding of human relationships. As such, this article will explore the associations between intimacy, olfactory ability, and disgust sensitivity.

### Intimacy and Olfaction

Intimacy is described as a feeling of closeness and connectedness with one’s partner (Laurenceau et al., 2005; Sternberg, 1997). Five forms of intimacy are broadly recognised (Schaefer & Olson, 1981)—emotional, physical, intellectual, social, and recreational. The existing research on these forms of intimacy and olfaction are discussed below.

#### Emotional Intimacy and Olfaction

Emotional intimacy embodies positive communication, validation, disclosure, and emotional reciprocity, which contribute to an overall feeling of closeness to one’s partner (Hook et al., 2011; Schaefer & Olson, 1981). Existing research supports a relationship between emotional intimacy and olfactory ability. For example, exposure to a partner’s body odor is associated with stress reduction (Granqvist et al., 2019; Hofer et al., 2018), while positively perceiving a partner’s body odor is correlated with greater relationship commitment (Keaveny & Mahmut, 2021). Additionally, reported relationship security is also higher in women with normative olfactory function compared to those with no sense of smell (Croy et al., 2013). Collectively, these findings highlight the potential role of olfactory ability in relationship satisfaction.

#### Physical Intimacy and Olfaction

Physical intimacy encompasses touch, physical closeness, and sexual activity (González-Rivera et al., 2019). Research has suggested physical intimacy’s role in intimate relationships with associations between better olfactory ability and increased sexual pleasure (Bendas et al., 2018), and more frequent sniffing a partner’s body odor with increased sexual desire (Li et al., 2022). This relationship is supported by Croy et al.’s (2013) finding that men with normative olfactory ability reported approximately five times more sexual relationships than men with no sense of smell (Croy et al., 2013). In sum, olfactory ability appears linked with physical intimacy and satisfaction in intimate relationships.

#### Intellectual, Social, and Recreational Intimacy and Olfaction

Minimal research has explored intellectual, social, and recreational forms of intimacy. However, Greeff and Malherbe (2011) reported positive correlations between these forms of intimacy and long-term relationship satisfaction. To date, the relationship between these forms of intimacy and olfactory ability or disgust has not been explored.

### Intimacy and Disgust

Disgust is a universal emotion and serves an evolutionary purpose of avoiding disease from harmful contaminants, such as faeces or spoiled food (Oaten et al., 2009). Disgust is commonly measured via disgust sensitivity, referring to the degree or intensity people experience disgust towards various stimuli (e.g. foul body odor; Massar, 2021; Tybur, 2021). While disgust serves as a mechanism for disease-avoidance, it is also an important emotion in interpersonal relationships, influencing sexual behaviour and social interactions (Rozin et al., 2000). This paper will focus on Tybur et al.’s (2009) three primary elicitors of disgust: pathogen, sexual and moral, reflected in their Three Domains of Disgust Scale and described in the following section.

#### Intimacy and Moral Disgust

Moral disgust pertains to social and moral transgressions, such as lying, cheating, and stealing (Simpson et al., 2006). Few studies have explored moral disgust in the context of romantic relationships; however, there are plausible theoretical links. For example, O’Shea et al. (2019) found those higher in moral disgust sensitivity were less likely to engage in short-term mating, albeit this association was noted as weak, and the significant result was attributed to the study’s large sample. Additionally, men who are higher in moral disgust sensitivity are more likely to engage in protective sexual behaviours, such as wearing condoms and testing for sexually transmitted diseases (Zhang et al., 2017). These studies suggest a potential relationship between intimacy and moral disgust. However, further research is needed to unpack this relationship and explore the potential association between intimacy and social transgressions such as infidelity in romantic relationships.

#### Intimacy and Sexual Disgust

Sexual disgust evolved to avoid reproductively costly sexual behaviours, such as suboptimal partners or unsafe sexual activity (Tybur et al., 2009). Prior research has suggested that couples with similar and lower sexual disgust sensitivities reported elevated sexual and marital satisfaction (Peters & Meltzer, 2021). Further, experimental studies have shown that sexual arousal can attenuate disgust in both males and females. Specifically, sexually aroused participants experienced less subjective disgust in response to sex-related disgust elicitors, than non-aroused participants (Borg & de Jong, 2012; Stevenson et al., 2011). A potential relationship between reduced sexual disgust sensitivity and increased interest in pursuing short-term mating (e.g., one-night stands) has also been suggested (Al-Shawaf et al., 2015; O’Shea et al., 2019; Pavela-Banai et al., 2021). Overall, there appears to be an association between sexual arousal, as well as mating strategy, and sexual disgust sensitivity.

#### Intimacy and Pathogen Disgust

Pathogen disgust is elicited by infectious agents, such as faeces and vomit, and motivates avoidance of such stimuli (Tybur et al., 2009). Studies have found that inducing disgust, for example, by exposing participants to images of genitalia with sexually transmitted infections, reduces desire for short-term mating (Al-Shawaf et al., 2015). Similar findings were reported by Zsok et al. (2017), who found that independent of sexual arousal, women reported greater disgust towards engaging in sexual contact with men exhibiting signs of disease (e.g. blemishes or scabs on face), indicating the presence of pathogens can affect appraisals of attractiveness and desirability. Those higher in pathogen disgust also report less interest in interpersonal affiliation (Sacco et al., 2014) and lower sexual arousal (Borg & de Jong, 2012). As such, the existing links between pathogen disgust and desire for short-term mating may suggest a similar relationship between pathogen disgust and intimacy, presumably physical intimacy.

### The Link Between Olfaction and Disgust

Olfaction and disgust both serve an evolutionary purpose of identifying harmful stimuli in our environment (Stevenson, 2010). Their relationship is well supported by existing literature. For example, odors perceived via our olfactory system appear to be pertinent stimuli in eliciting disgust reactions (Syrjänen et al., 2017), and disgust and olfactory cues also provide important cues regarding the health and emotional status of others (Mahmut & Croy, 2019; Oaten et al., 2009). However, olfaction and disgust have not yet been studied in conjunction with intimacy.

### The Link Between Olfaction, Disgust, and Intimacy

Olfaction, disgust, and intimacy will now be discussed together. Although these three variables have not been studied together, preliminary research supports the connection. For example, Blomkvist et al. (2021) found pathogen disgust mediated the relationship between self-reported olfactory function and couple’s sexual well-being (e.g. satisfaction, arousal). That is, olfactory function predicted sexual well-being via pathogen disgust. Additionally, Prokosch et al. (2021) found sexual disgust mediated the relationship between odor acuity and short-term mating strategies; that is, those with greater ability to discriminate odors demonstrated higher sexual disgust and were less inclined to engage in short-term mating (e.g. one-night stands).

Despite the limited empirical basis, there are theoretical links between intimacy, olfaction, and disgust. As previously summarised, both disgust and olfaction motivate avoidance of contaminating substances (Croy et al., 2014; Oaten et al., 2009). This avoidance mechanism also transcends into interpersonal relationships, where disease and emotion cues of others (exhibited by body odors, facial expressions, and overall appearance) flag who we wish to become close to or avoid (Olsson et al., 2014; Zsok et al., 2017). Such cues may subsequently provide important signals about an optimal mate to develop a close, intimate relationship with (Havlicek et al., 2008; Mahmut & Croy, 2019; Tybur et al., 2009). Additionally, olfactory impairments may impede fulfilling, romantic relationships by depriving individuals of the emotional reciprocity and security that come from a partner’s smell (Blomkvist & Hofer, 2021). Similarly, high disgust sensitivity can dampen sexual arousal and desire (de Jong et al., 2013) in turn potentially impacting couple’s long-term sexual routine and satisfaction (Leavitt & Willoughby, 2014; McNulty et al., 2014). Therefore, high disgust sensitivity and poor olfactory ability may affect overall relationship and sexual satisfaction, which underpin intimacy.

### The Current Study

The aim of the present study is to examine the association between intimacy, olfactory ability, and disgust sensitivity. In doing so, three lesser studied types of intimacy will be explored in addition to emotional and physical: intellectual, recreational, and social. In addition, the present study will expand on previous findings by including participants who are presently single but have previously been partnered, allowing for exploration of experiences related to relationship breakdown.

Three predictions were made based on existing literature (Bendas et al., 2018; Blomkvist & Hofer, 2021). First, greater olfactory ability will be associated with greater emotional intimacy. Second, greater olfactory ability will also be associated with greater physical intimacy. Third, greater pathogen and sexual disgust will be negatively associated with physical intimacy.

## Method

### Participants

Participants were recruited in one of three ways: (1) voluntarily via an online recruitment system, SONA, (2) via email invitation if they completed a screener survey, or (3) via flyers distributed on Macquarie University campus and social media. Participants either received course credit or went into a draw to win one of three $50 vouchers of their choosing, as reimbursement for completing the study.

Only participants who are currently, or have previously been, in a romantic relationship were invited to complete the study. One participant who had never been in a romantic relationship completed the study and was therefore excluded. Additionally, three participants failed embedded attention checks and were excluded.

The final example consisted of 74 participants (20 males, 51 females, three non-binary) with age ranging from 17 to 56 years (*M* = 22.6, *SD* = 7.1). Sixty-one participants identified as heterosexual, three identified as gay or lesbian, nine identified as bisexual, and one participant did not specify. Of these participants, 56 participants were currently partnered (*M*_age_ = 23.1, *SD*_age_ = 7.9) and 18 were currently single but had previously been in a relationship (*M*_age_ = 21.3, *SD*_age_ = 2.9), referred to as single, previously partnered in the remainder of the article. The length of romantic relationships ranged from three months or less to over five years, with a median length of 1–3 years. The number of romantic relationships experienced by participants ranged from 1–9 *(M* = 2.0, *SD* = 1.4). Most participants had engaged in sexual activity (*n* = 66), with five reporting that they had not, and three selecting “prefer not to say.”

An a priori power analysis was conducted via G*Power 3.1. For 90% power at .05 significance, 61 participants were sufficient to detect a small to moderate effect size of *r* = .40. As such, adequate statistical power was achieved with the study’s sample size of 74 participants.

### Measures

#### Personal Assessment of Intimacy in Relationships

The Personal Assessment of Intimacy in Relationships (PAIR; Schaefer & Olson, 1981) is a 36-item self-report scale, designed to measure intimacy in romantic relationships and has five subscales: emotional, physical, intellectual, social, and recreational. A conventionality scale is also included to determine if participants are providing socially desirable answers. Six items are included for each subscale, with response options on a 5-point Likert scale (1 = *does not describe me/my relationship at all*, 5 = *describes me/my relationship very well).* Total scores for each subscale were created by summing responses (six items were reverse-scored), with higher scores indicating higher self-reported intimacy. The emotional, physical, and intellectual subscales of the PAIR have acceptable internal consistency in previous studies (*α* < .70; Schaefer & Olson, 1981). In the present study, emotional, physical, and intellectual intimacy display acceptable internal consistency (emotional, *α* = .89; physical, *α* = .78; intellectual), while social and recreational intimacy demonstrated lower internal consistency (social, *α* = .56; recreational, *α* = .66).

#### Three Domains of Disgust Scale

The Three Domains of Disgust Scale (TDDS; Tybur et al., 2009) is a 21-item self-report measure of disgust in three domains: moral disgust (e.g. stealing from a neighbour); sexual disgust (e.g. performing oral sex) and pathogen disgust (e.g. stepping on dog poo). Each subscale has seven items and participants rate how disgusting they find the described concept using a 7-point scale (0 = *not at all disgusting*, 6 = *extremely disgusting*). Items for each subscale were summed to create an overall moral, sexual, and pathogen score, respectively, with higher scores indicating greater levels of disgust. The TDDS has demonstrated adequate reliability in previous studies (moral, *α* = .91; sexual, *α* = .86; pathogen, *α* = .80; Tybur et al., 2009), and the current study (moral, *α* = .87; sexual, *α* = .77; pathogen, *α* = .70).

#### Sniffin’ Sticks Test (Short Form)

The five-item *Sniffin’ Sticks* test (Mueller & Renner, 2006) is a shortened form of the 16-item *Sniffin’ Sticks* identification test (Hummel et al., 1997). The test measures olfactory ability via correct identification of odors and adequately correlates with the 16-item measure (*r* = .61, *p* < .01; Mueller & Renner, 2006). Odorants were presented in 30 mL plastic jars with a screw-top lid. Four millilitres of rose (Odor A), peppermint (Odor B), orange (Odor C), and leather (Odor D) odors were placed separately in opaque, individual jars. Fish (Odor E) was in solid form, using 3 g of benito flakes. To smell the odor, the experimenter demonstrated to participants to bring the jar 2 cm away from their nostrils and sniff twice. Participants smelled each odor in alphabetical order and were presented with 20 different response items (5 correct responses and 15 distractors) and instructed to select one correct response for each odor. Scores ranged from 0 to 5, with higher scores indicating greater olfactory ability. Odors were refrigerated at a temperature of approximately 5 °C outside of testing periods to maintain freshness and removed approximately 30 min prior to testing. The short form *Sniffin’ Sticks* test has demonstrated adequate test–retest reliability (*r* = .77, *p* < .01; Mueller & Renner, 2006). While internal consistency was not identified in Mueller and Renner’s (2006) study, it was acceptable for the present study (α = .78)*.*

### Procedure

The study took place in a Macquarie University laboratory over a one-hour session. Upon arrival, participants were asked if they were experiencing any cold or flu-like symptoms that may affect their sense of smell and ability to participate in the study. Once no symptoms were confirmed, participants were then emailed a URL link to complete the study, which was hosted on the online platform Qualtrics. After reading an information form outlining details of the study and providing informed consent to participate, participants then answered demographic questions and completed the PAIR, TDDS, and *Sniffin’ Sticks* test in this order. After completing the study, participants were verbally debriefed about the study’s aims and were excused.

### Analysis Plan

Results are presented in three sections. First, descriptive statistics are presented. Second, bivariate analyses were conducted to assess potential confounds and examine relationships. As multiple key variables were significantly non-normal (Shapiro–Wilk *p* > .001), Spearman Rank-Order correlations and Kruskal–Wallis were used for bivariate analyses. Bivariate analyses conducted using the Kruskal–Wallis H Test all featured follow-up comparisons using Dunn’s test with a Bonferroni adjustment. Third, five bootstrapped regression analyses with 2000 repetitions were run to explore whether key variables could predict changes in intimacy. The alpha level was set to .05. Although non-binary participants were retained in the sample, significant results are not interpreted due to the insufficient sample size. Data were analyzed using STATA MP (version 17.0), and alpha was set at .05 for all analyses.

## Results

### Descriptive Statistics

Descriptive statistics of key variables are displayed in Table 1.

**Table 1:** Descriptive statistics (*N* = 74)

Variables	Mean	SD	Min	Max
*Intimacy*				
Emotional	24.6	5.1	9	30
Physical	23.4	4.8	7	30
Intellectual	22.7	2.8	16	28
Social	20.6	3.1	14	27
Recreational	21.4	3.1	14	27
Olfactory ability	4.0	1.1	1	5
*Disgust*				
Moral	23.4	7.0	6	41
Sexual	16.5	8.0	0	36
Pathogen	25.9	8.9	1	40

### Bivariate Analyses

Correlational analyses are presented in Table 2. In line with prediction, there was a significant positive correlation between olfactory ability and emotional intimacy, albeit weak. Contrary to prediction, there was no significant correlation between olfactory ability and physical intimacy. Also in contrast to prediction, weak positive (rather than negative) correlations were found between sexual disgust and physical intimacy, and pathogen disgust and physical intimacy.

**Table 2:** Spearman’s rank-order correlation matrix (*N* = 74)

Variables	1	2	3	4	5	6	7	8	9	10	11	12
1. Emotional intimacy	–											
2. Physical intimacy	.52***	–										
3. Intellectual intimacy	.70***	.35**	–									
4. Social intimacy	.41***	.21	.32**	–								
5. Recreational intimacy	.58***	.20	.57***	.22	–							
6. Olfactory ability	.24*	.07	.23*	.04	.20	–						
7. Moral disgust	.12	.20	− .05	− .05	− .04	.07	–					
8. Pathogen disgust	.11	.24*	.06	.39***	− .14	.11	.19	–				
9. Sexual disgust	.15	.24*	.12	.08	.05	.29*	.42***	.25*	–			
10. Relationship status^a^	.58***	.32**	.47***	.29*	.37**	.01	− .06	− .18	.05	–		
11. Relationship length^b^	.15	.09	.20	.30**	.12	.19	− .17	.01	− .05	.46***	–	
12. No. of relationships	− .09	.02	.12	.04	− .10	.06	− .07	.11	− .19	− .04	.21	–
13. Age	− .09	− .16	.07	.20	− .01	.09	− .05	− .02	− .26*	.05	.56***	.37***

^a^0 = single, 1 = partnered

^b^0 = 3 months or less, 1 = 3–6 months, 2 = 6–12 months, 3 = 1–3 years, 4 = 3–5 years, 5 = 5 + years

*p < .05, **p ≤ .01, ***p ≤ .001

The exploratory analyses also revealed a significant weak positive correlation between intellectual intimacy and olfactory ability. However, there was no relationship between social or recreational intimacy and olfactory ability. In addition, there was a significant weak correlation between physical intimacy and both pathogen and sexual disgust. There was also a moderate positive correlation between social intimacy and pathogen disgust. Moral disgust was not related to any variable of interest. No variables of interest were related to recreational intimacy.

Potential covariates were identified. All forms of intimacy were significantly higher for partnered participants compared to single participants. Relationship length was also significantly moderately positively correlated with social intimacy.

There was a significant effect of gender on numerous key variables, including physical intimacy, *H*(2) = 11.15, *p* = .004, with intimacy significantly higher in females (*Mdn* = 25.0) compared to males (*Mdn* = 21.0), *p* < .001). There was no effect of gender on emotional, intellectual, social, or recreational intimacy.

There was also a significant effect of gender on olfactory ability, *H*(2) = 10.88, *p* = .004, with females (*Mdn* = 4.0) demonstrating better olfactory ability than males (*Mdn* = 3.5, *p* = .003). While there was no effect of gender on moral or pathogen disgust, there was a significant effect on sexual disgust, *H*(2) = 22.39, *p* < .001, with sexual disgust higher in females (*Mdn* = 27.0) compared to males (*Mdn* = 23.0, *p* < .001).

### Regression Analyses

Five bootstrapped regression analyses were run with each type of intimacy as the dependent variable to unpack bivariate findings. Gender, relationship status, and relationship length were included as covariates due to significant bivariate relationships with at least one of the five forms of intimacy. Results are displayed in Table 3.

**Table 3:** Regression analyses (*N* = 74)

Emotional intimacy	*b*	SE	95% CI (BC)	*p*
Olfactory ability	.57	.47	[− .26, 1.61]	.23
Moral disgust	.07	.06	[− .03, .22]	.24
Sexual disgust	.02	.08	[− .14, .18]	.79
Pathogen disgust	.05	.05	[− .05, .15]	.32
Gender				
Female (vs. male)	− .90	1.17	[− 3.26, 1.35]	.44
Male (vs. non-binary)	− .20	4.16	[− 5.79, 10.75]	.63
Non-binary (vs. female)	2.91	4.13	[− 11.16, 5.04]	.48
Relationship status^a^	8.86	1.41	[6.30, 11.91]	< .001
Relationship length^b^	− .63	.57	[− 1.60, .29]	.18

Physical intimacy	*b*	SE	95% CI (BC)	*p*
Olfactory ability	− .54	.43	[− 1.42, .28]	.21
Moral disgust	.10	.06	[− .01, .25]	.10
Sexual disgust	− .03	.07	[− .17, .11]	.68
Pathogen disgust	.13	.05	[.04, .25]	.009
Gender				
Female (vs. male)	4.03	1.35	[1.48, 6.84]	.003
Male (vs. non-binary)	2.11	5.21	[− 9.65, 10.20]	.69
Non-binary (vs. female)	− 6.14	5.26	[− 6.02, 14.22]	.24
Relationship status^a^	3.01	1.43	[.13, 5.72]	.035
Relationship length^b^	.46	.56	[− .49, 1.71]	.42

Intellectual intimacy	*b*	SE	95% CI (BC)	*p*
Olfactory ability	.59	.30	[− .01, 1.17]	.053
Moral disgust	− .03	.04	[− .10, .05]	.42
Sexual disgust	.01	.05	[− .09, .10]	.91
Pathogen disgust	.02	.03	[− .04, .09]	.45
Gender				
Female (vs. male)	.05	.75	[− 1.43, 1.59]	.95
Male (vs. non-binary)	− .65	1.48	[− 2.60, 3.43]	.66
Non-binary (vs. female)	.60	1.50	[− 3.31, 2.58]	.69
Relationship status^a^	3.58	.88	[1.84, 5.29]	< .001
Relationship length^b^	− .21	.35	[− .93, .47]	.54

Social intimacy	*b*	SE	95% CI (BC)	*p*
Olfactory ability	− .01	.35	[− .69, .68]	.99
Moral disgust	− .03	.05	[− .12, .06]	.54
Sexual disgust	.02	.05	[− .09, .11]	.68
Pathogen disgust	.13	.04	[.04, .22]	.003
Gender				
Female (vs. male)	− .21	.92	[− 2.03, 1.60]	.82
Male (vs. non-binary)	− .06	1.63	[− 3.18, 3.37]	.97
Non-binary (vs. female)	.26	.02	[− 3.42, 3.11]	.87
Relationship status^a^	2.52	.96	[.65, 4.41]	.009
Relationship length^b^	.29	.29	[− .25, .87]	.32

Recreational intimacy	*b*	SE	95% CI (BC)	*p*
Olfactory ability	.76	.33	[.14, 1.42]	.019
Moral disgust	− .02	.06	[− .12, .11]	.74
Sexual disgust	− .02	.06	[− .14, .09]	.701
Pathogen disgust	− .03	.04	[− .11, .07]	.49
Gender				
Female (vs. male)	.02	.84	[− 1.76, 1.62]	.98
Male (vs. non-binary)	2.52	2.39	[− 7.69, 1.75]	.29
Non-binary (vs. female)	− .25	2.42	[− 1.73, 7.68]	.29
Relationship status^a^	2.64	.88	[.92, 4.34]	.003
Relationship length^b^	− .31	.31	[− .89, .35]	.32

*b*, Unstandardised beta; SE, standardised error; CI, confidence intervals; BC, bias corrected. SEs and significant levels are bootstrapped

^a^0 = single, 1 = partnered

^b^0 = 3 months or less, 1 = 3–6 months, 2 = 6–12 months, 3 = 1–3 years, 4 = 3–5 years, 5 = 5 + years

All five models were significant. Relationship status emerged as a significant unique predictor in all models, with currently partnered participants demonstrated significantly higher intimacy compared to single but previously partnered participants. Although the model predicting emotional intimacy was significant (Wald $\chi$ ^2^(8) = 54.66, *p* < .001, *R*^2^ = 47.42%) there were no other unique predictors.

In addition to relationship status, pathogen disgust, and gender uniquely predicted physical intimacy (Wald $\chi$ ^2^(8) = 33.72, *p* < .001, *R*^2^ = 37.30%). Specifically, as pathogen disgust increased, so did physical intimacy. Physical intimacy was also significantly higher in females compared to males. In the model predicting intellectual intimacy (Wald $\chi$ ^2^(8) = 31.27 *p* < .001, *R*^2^ = 31.27%), olfactory ability was borderline significant (*p* = .053) with intellectual intimacy increasing as olfactory ability increases. The model predicting social intimacy (Wald $\chi$ ^2^(8) = 23.38, *p* = .003, *R*^2^ = 26.58%) featured pathogen disgust as a significant unique predictor. Specifically, as pathogen disgust increases, so does social intimacy. Finally, in the model predicting recreational intimacy (Wald $\chi$ ^2^(8) = 22.93, *p* = .004, *R*^2^ = 23.56), olfactory ability emerged as a significant unique predictor.

## Discussion

The primary aim of this study was to investigate the association between olfactory ability, disgust sensitivity, and emotional and physical intimacy. One of three predictions was supported, with a positive relationship between olfactory ability and emotional intimacy. However, this relationship was weak and did not remain after controlling for covariates. In contrast to prediction, sexual and pathogen disgust were positively related to physical intimacy. These findings, as well as the exploratory findings, will be discussed in the context of previous findings. The Discussion will then outline the study’s strengths and limitations, implications, and directions for future research, before closing with a summary of the present study.

### Was Emotional Intimacy Related to Olfactory Ability?

Although olfactory ability initially positively correlated with emotional intimacy, the relationship was weak and not significant after controlling for covariates. This conflicts with previous research linking olfactory ability with enhanced interpersonal relationships (Blomkvist & Hofer, 2021; Croy et al., 2012; Keaveny & Mahmut, 2021). A potential explanation for this finding is the inclusion of previously partnered participants in the sample, who reported significantly lower intimacy than partnered participants.

### Was Physical Intimacy Related to Olfactory Ability?

Olfactory ability was not related to greater physical intimacy. This contradicts previous findings suggesting that greater olfactory ability is associated with enriched sexual experiences (Croy et al., 2009, 2013). For example, olfaction has been linked to arousal, desire, and sexual satisfaction, with prior studies finding those that assign greater value to olfaction and sniff their partner’s body odors experience increased sexual desire (Li et al., 2022). Additionally, those higher in olfactory ability are more likely to have greater sexual experience, pleasure, and more frequent orgasms (Bendas et al., 2018). A possible explanation for this is our sample’s heavy skew towards female participants. This may contribute to our findings as past research suggests men place greater emphasis on physical and sexual aspects of one’s relationship, whereas women preference emotional connection and communication (Hook et al., 2011; Sprecher, 2002; van Lankveld et al., 2018). The opposite was found in our sample, with females having significantly higher physical intimacy than males, indicating insufficient data and variability in responses from males. As such, the non-significant association between olfactory ability and physical intimacy may be attributed to a gender imbalance.

### Was Physical Intimacy Related to Disgust Sensitivity?

Pathogen and sexual disgust were positively, rather than negatively, associated with physical intimacy. Only the relationship between pathogen disgust and physical intimacy remained in the full regression model. In contrast, prior research has found couples lower in sexual disgust report greater sexual satisfaction (Peters & Meltzer, 2021) and higher pathogen disgust is associated with less interpersonal affiliation (Sacco et al., 2014). In addition, research demonstrates pathogen and sexual disgust have inhibitory effects on arousal and desire for short-term mating (Al-Shawaf et al., 2015; Borg & de Jong, 2012; Zsok et al., 2017). The discrepancy between past and current findings may be partially explained by some studies focusing on desire for short-term mating as the variable of interest, rather than intimacy more generally (Al-Shawaf et al., 2015). Additionally, the current study measured physical intimacy, rather than arousal, with questions asking about satisfaction of one’s sex life, mutual interest in sex, and sexual expression.

### Exploratory Analyses: Intellectual, Social, and Recreational Intimacy, and their Relationship with Olfaction and Disgust

Exploratory analyses revealed a positive relationship between intellectual intimacy and olfactory ability, albeit weak, as well as a positive relationship between social intimacy and pathogen disgust. Interestingly, olfactory ability emerged as significant unique predictor of recreational intimacy. Further research is needed to explore the relationship between intellectual, social, and recreational intimacy with olfaction and disgust in a larger sample of partnered participants.

### Strengths and Limitations

The present study has several strengths. Notably, this study is of the small minority that investigated disgust in a committed, monogamous context, and subsequently found an association between greater pathogen and sexual disgust and increased physical intimacy. As most disgust research focuses on desire for short-term mating, the current study adds to our understanding of mating strategies and relationships. Additionally, it expands research on intimacy, by investigating intellectual, social, and recreational intimacy in relation to olfaction and disgust.

The study also has limitations. The main limitation is the use of a five-item measure of *Sniffin’ Sticks* odor identification test, resulting in low variability in olfactory ability scores and a high mean score, which may have increased Type II error. Additionally, the correlational nature of the study means causal inferences cannot be made. Other notable limitations include the sample’s skew towards younger, female participants, and a lack of detailed health screening before olfactory testing.

### Implications and Directions for Future Research

To enhance the validity and generalisability of these preliminary findings, future research is needed. Studies should employ the full 16-item *Sniffin’ Sticks* odor identification test and include additional olfactory tests on threshold and discrimination. Additionally, comprehensive health screenings could help in identifying confounding variables. Further studies should also aim for greater sample diversity, particularly in terms of gender, sexual orientation, and relationship type (e.g. monogamous, polyamorous). Exploring different demographics will deepen our understanding of how disgust and olfaction interact with intimacy across different contexts.

### Conclusion

The present study is the first to examine intimacy, disgust, and olfactory ability together. In doing so, the findings contribute to a deeper understanding of variables impacting romantic relationships. While the preliminary findings suggest significant but weak associations between the variables of interest, replication with larger samples is needed. Additionally, the generalisability of the current findings should be explored in future studies by broadening the diversity of the participants in terms of age and relationship type.

## Data Availability

The data that support the findings of this study are available from the corresponding author, Fiona E. Wylie, upon reasonable request.
